# The Nature of Ferromagnetism in a System of Self-Ordered α-FeSi_2_ Nanorods on a Si(111)-4° Vicinal Surface: Experiment and Theory

**DOI:** 10.3390/nano12203707

**Published:** 2022-10-21

**Authors:** Nikolay G. Galkin, Dmitrii L. Goroshko, Ivan A. Tkachenko, Aleksey Yu. Samardak, Konstantin N. Galkin, Evgenii Yu. Subbotin, Sergei A. Dotsenko, Dmitry B. Migas, Anton K. Gutakovskii

**Affiliations:** 1 Laboratory of Optics and Electrophysics, Institute of Automation and Control Processes, FEB RAS, Radio Str. 5, 690041 Vladivostok, Russia; 2 Laboratory of Chemical Radiospectroscopy, Institute of Chemistry, FEB RAS, Pr. 100th Anniversary of Vladivostok, 159, 690022 Vladivostok, Russia; 3 Film Technology Laboratory, Far Eastern Federal University, Russky Island, FEFU Campus, Building L, 690922 Vladivostok, Russia; 4 Department of Micro- and Nanoelectronics, Belarusian State University of Informatics and Radioelectronics, P. Browka 6, 220013 Minsk, Belarus; 5 Moscow Engineering Physics Institute, National Research Nuclear University “MEPhI”, Kashirskoe Shosse 31, 115409 Moscow, Russia; 6 Rzhanov Institute of Semiconductor Physics SB RAS, Pr. ak. Lavrentiev, 13, 630090 Novosibirsk, Russia

**Keywords:** vicinal silicon surface, iron disilicide, self-ordered growth of nanorods, magnetization reversal loops, ab initio magnetic calculations, nature of nanorod ferromagnetism

## Abstract

In this study, the appearance of magnetic moments and ferromagnetism in nanostructures of non-magnetic materials based on silicon and transition metals (such as iron) was considered experimentally and theoretically. An analysis of the related literature shows that for a monolayer iron coating on a vicinal silicon surface with (111) orientation after solid-phase annealing at 450–550 °C, self-ordered two-dimensional islands of α-FeSi_2_ displaying superparamagnetic properties are formed. We studied the transition to ferromagnetic properties in a system of α-FeSi_2_ nanorods (NRs) in the temperature range of 2–300 K with an increase in the iron coverage to 5.22 monolayers. The structure of the NRs was verified along with distortions in their lattice parameters due to heteroepitaxial growth. The formation of single-domain grains in α-FeSi_2_ NRs with a cross-section of 6.6 × 30 nm^2^ was confirmed by low-temperature and field studies and FORC (first-order magnetization reversal curves) diagrams. A mechanism for maintaining ferromagnetic properties is proposed. Ab initio calculations in freestanding α-FeSi_2_ nanowires revealed the formation of magnetic moments for some surface Fe atoms only at specific facets. The difference in the averaged magnetic moments between theory and experiments can confirm the presence of possible contributions from defects on the surface of the NRs and in the bulk of the α-FeSi_2_ NR crystal lattice. The formed α-FeSi_2_ NRs with ferromagnetic properties up to 300 K are crucial for spintronic device development within planar silicon technology.

## 1. Introduction

The issue of induced magnetism in nanostructures (NSs) of non-magnetic materials such as iron mono (FeSi) and disilicides (FeSi_2_) has attracted much attention from experimenters [1,2,3,4,5,6] and theorists [7,8,9,10]. The appearance of ferromagnetism (FM) and superparamagnetism (SPM) in NSs from a theoretical point of view can be explained either by a change in a number of first neighbors and interatomic distances of iron atoms in the bulk crystal structure [7] or by the presence of Fe atoms at the edges of the NS, which have nonzero spin moments and are a source of magnetism [8]. In ultrathin films of FeSi, as theoretically established in [9], FM is determined by the upper layer in the FeSi structure (i.e., by termination). When the FeSi layer is terminated by iron atoms, a strong ferromagnetic moment is observed (2.6 Bohr magneton per Fe atom), which sharply decreases (0.2 Bohr magneton per Fe atom) when the FeSi layer is terminated by Si atoms. Even though the SPM phenomenon is often observed experimentally for magnetic NSs of various compositions when investigating the temperature dependence of the magnetic moment both in and without a magnetic field, it still requires a theoretical understanding. A study in this direction was reported in [10], in which analytical expressions were derived for the magnetic moment with and without cooling in a magnetic field for a system of polydisperse magnetic particles. Such a system is closer to the experimentally observed particle size distribution and can be used to fit experimental data for SPM systems. Regarding the self-organized formation of such NSs (nanowires (NWs) and nanorods (NRs)) of silicides of transition and rare earth metals on silicon, one can use the deposition of a small amount of metal at room temperature with the following annealing at different temperatures. In particular, silicides can be grown with an anisotropy of the mismatch of crystal parameters with silicon in mutually perpendicular directions [1,11] and iron disilicides can be grown with different crystal structures [2,3,4] on silicon vicinal surfaces with step-bunches, which do not exhibit ferromagnetic properties in the bulk [12].

It was reported that, during ultrahigh vacuum (UHV) deposition of one iron monolayer (ML) onto the Si(110) at 700 °C NWs of the metastable cubic γ-phase of iron disilicide (γ-FeSi_2_) were formed with dimensions of 5 × 10 × (1000 ÷ 2000) nm due to small lattice mismatch with silicon [1]. A ferromagnetic hysteresis loop with a coercive force of approximately 30 Oe was identified in a magnetic field parallel to the NW axis at 2 K. This was close to the SPM phenomenon, taking into account the number of deposited iron atoms corresponding to a magnetic moment of 0.3 ± 0.1 Bohr magneton per Fe atom. However, no reason for the appearance of induced magnetism was proposed by the authors.

UHV studies of the solid-state growth, when depositing iron atoms with different coatings on a Si(111)-4° vicinal surface at different temperatures [2], showed that, at a low iron coating (1.2 MLs) and annealing temperatures ranging from 450 °C to 550 °C, formation of elongated islands (self-ordering NRs) of α-FeSi_2_ occurred, which were oriented along the step-bunches. The density of α-FeSi_2_ islands depended on the amount of iron and annealing temperature. When the iron coating was increased to 21 MLs and annealed at 700 °C, three-dimensional disordered islands of α-FeSi_2_ were formed [2]. Both types of islands exhibited ferromagnetic hysteresis loops at 4 K with different coercive forces displaying their maximum in a magnetic field directed in the island plane (in-plane) according to the chain alignment model [2] and their minimum—in a plane perpendicular to the substrate and islands (out-of-plane). According to the SQUID data [2], SPM properties emerged in a non-magnetic material with a high total magnetic moment of 1.9 Bohr magnetons per Fe atom at a low Fe coverage (1.2 MLs) and 0.9 Bohr magnetons per Fe atom at a Fe coverage of 21 MLs. The authors attributed these properties to the presence of Fe-enriched defects in the α-FeSi_2_ lattice detected by XPS data, whereas the high SPM blocking temperature (approximately 225 K) was associated with the large size of the nanoislands and their anisotropy [2].

An additional study of α-FeSi_2_ NRs on a vicinal Si(111)-4° surface, formed under UHV conditions with a single annealing of 1 Fe ML at 550 °C [3], analyzed the anisotropy of the magnetic properties along and across the NRs as a function of temperature. At room temperature, the coercive force along the NRs was equal to zero, indicating the SPM nature of magnetization. With decreasing temperature, the coercive force increased to 35 Oe at 150 K and 212 Oe at 4 K, demonstrating classical ferromagnetic behavior. In this case, at 4 K, the coercive force along the NRs was more than three times higher than that across the NRs. A similar anisotropy of the coercive force was previously observed along other ferromagnetic NWs [13,14]. This issue is associated with more difficult magnetization reversal along the NWs [2]. The observed difference in the magnetic behavior of α-FeSi_2_ NRs (quasi-one-dimensional islands) and three-dimensional α-FeSi_2_ islands, according to [2,3], is that defects enriched in iron atoms are generated along, rather than across, the NWs, leading to a larger induced magnetic moment along the NWs at low iron coverages. It was reported in [2,3] that, depending on the iron coverage and annealing temperature, both the shape of the islands and their size and density on the surface change that is critical for manufacturing ultrahigh density magnetic memory.

In connection with the last statement in [4], on vicinal surfaces of Si(111)-4° and Si(001)-2° under UHV annealing conditions, the formation of epitaxial nanoislands of iron silicides at various iron coatings (0.5 MLs, 1–2 to 2–10 MLs) and annealing temperatures (from 500 °C to 750 °C) was studied. Islands with different sizes, densities, and with and without self-organization formation were identified. It was found that with submonolayer and monolayer coverages, formation of γ-FeSi_2_ islands was observed, and an increase in the iron coverage to 10 MLs and annealing at 500 °C led to the formation of an almost continuous silicide film consisting of two types of islands (γ-FeSi_2_ and ε-FeSi). Studies on magnetization reversal loops with a magnetic field in the plane of the substrate showed that the magnetic response was superparamagnetic with zero or low (100–400 Oe) coercive force at 300 K and 4 K for samples with different iron coatings on Si(001)-2° and Si(111)-4° substrates. An anomalous coercive force of up to 2 kOe was observed in a sample coated with iron from 1 ML to 2 MLs on a Si(111)-4° substrate annealed at 550 °C. By analogy with [2,3], it is suggested [4] that iron atoms with a smaller number of first neighbors at the edges of γ-FeSi_2_ and ε-FeSi islands provide the main contribution to the magnetic moment because defects are formed in places with the broken lattice symmetry [15,16]. For self-organizing nanoislands, these places are their edges and corners [8,17,18,19]. Therefore, the coercive force measured along the easy magnetization axis is a suitable parameter for explaining the magnetic anisotropy of nanoislands [20,21,22,23]. Using this parameter, it is concluded [4] that the magnetization loops in grown samples with iron silicide films do not depend on the orientation of the substrate but on the increase in the length-to-width aspect ratio in the formed islands. This ratio has the maximum for nanoislands in the sample (Si(111)-4°, 1–2 MLs of Fe, annealing at 550 °C, *Hc* = 2000 Oe) with a unique multi-terraced shape with many edges justifying a dipole–dipole interaction between SPM nanoislands.

Experimental studies of the resulting ferromagnetic properties were also conducted for nanoparticles, NWs, based on iron disilicide, including semiconducting β-FeSi_2_. β-FeSi_2_ NWs synthesized on Si(100) substrates by chemical vapor deposition [5] in the temperature range of 750–950 °C showed ferromagnetic behavior at room temperature with good field emission, in contrast to bulk and thin-film samples of β-FeSi_2_. The synthesized FeSi_2_ nanoparticles with sizes ranging from 15 to 55 nm demonstrated SPM properties [6] with different blocking temperatures depending on the size of the nanoparticles. It was found that a certain number of Fe ions appearing during the synthesis of FeSi_2_ nanoparticles and having other Fe atoms (ions) as nearest neighbors, are responsible for the ferromagnetic properties. When nanoparticles are cooled below 10 K, an anomaly of properties is observed in the form of spin glass, which also indicates the inhomogeneity of the nanoparticle composition. The structure and magnetic properties of samples with metallic iron nanocrystals and iron disilicide films after deposition of 5-nm-thick Fe layers on a Si(111) substrate and annealing (solid-phase epitaxy (SPE) process) were studied in [12]. At temperatures from 200 to 550 °C, only the formation of crystalline or polycrystalline Fe, which exhibited ferromagnetic properties, was detected. At an annealing interval from 5 to 200 min and temperature of 600 °C, the formation of the β-FeSi_2_ semiconductor phase was observed. It was shown that the ferromagnetic properties of a single-phase sample with β-FeSi_2_ crystals were not completely fixed, and in the case of a two-phase sample, they were determined by the contribution of iron nanocrystals. It was also established that chemically synthesized nanoparticles of β-FeSi_2_ have no ferromagnetic properties [6,24]. The manifestation of ferromagnetic properties in β-FeSi_2_ NWs reported in a number of studies [5,25] is apparently due to the lack of analysis of the composition and introduction of impurities in the form of iron atoms, which have been found in β-FeSi_2_ nanoparticles [12,26].

We have previously found a possibility to form self-ordered NRs of α-FeSi_2_ formed by SPE during one-stage 20 min annealing at 630 °C (after deposition of 10 Fe MLs on a Si(111)-3° with high n-type conductivity) under UHV conditions [27]. According to transmission electron microscopy (TEM) data of the cross-section, these NRs were compressed (−1.19%) along the “a” axis and stretched (12.98%) along the “c” axis, thereby increasing the unit cell volume of α-FeSi_2_ by 10.3%. The revealed distortions of the crystal lattice in α-FeSi_2_ NRs can provide the appearance of additional Fe atoms, which can cause changes in the magnetic properties of NSs, as reported in [2,3,4,6]. Formed self-ordered α-FeSi_2_ NRs [27] refer to the Fe coverage as an intermediate case compared to [2] but with a higher annealing temperature (630 °C) during the SPE process, which affects their size and density. However, the magnetic properties of these α-FeSi_2_ NRs with an intermediate coverage of iron atoms have not been studied to date, which indicates the relevance of this study.

## 2. Materials and Methods

### 2.1. Growth Chamber and Parameters

The growth of the iron disilicide was carried out by SPE in a Varian UHV chamber with a base pressure of 2 × 10^−10^ Torr equipped with a three-sample holder, quartz thickness gauge, and sublimation iron source. The substrate was a single-crystal silicon with (111) orientation and a misorientation angle of 4° (vicinal Si(111)-4° surface). An atomically clean sample surface was obtained using a series of high-temperature and short-term annealings at 1150 °C that were previously described in detail for the singular Si(111) [28] and high-index Si(55 12) [29] surfaces. The sample temperature was controlled using a PhotriX ML-AAPX infrared pyrometer. A rectangular silicon wafer (5 × 17 mm^2^) with p-type conductivity and a specific resistance of (0.002–0.0025) Ω × cm was used as the substrate. The rate of Fe deposition on the quartz thickness gauge was 0.12 nm/min. Iron disilicide NRs were formed via Fe deposition for 5 min on an atomically clean substrate at room temperature, followed by annealing at 630 °C for 20 min. Additional calibration of the metal coating (Fe) was carried out by depositing a thick metal film (approximately 50–70 nm) at a selected source current through a contact mask, followed by the determination of the film thickness by atomic force microscopy (AFM) and comparison of a similar portion of the metal deposited on a quartz thickness gauge. This made it possible to adjust the proportionality coefficient for a more accurate determination of the metal deposition rate.

### 2.2. Surface Morphology Characterization

The heterostructure morphology of the formed disilicide on the Si(111)-4° substrate was studied by AFM using a Solver P47 microscope after unloading the sample from the growth chamber in the semi-contact mode as well as after temperature magnetic measurements. AFM images were analyzed using a specialized program [30].

### 2.3. Crystal Structure Characterization 

The study of the crystal structure of Fe disilicide NR/Si(111)-4° hetero system was performed using a JEOL-4000EX (JEOL Ltd., Tokyo, Japan), which is a high-resolution transmission electron microscope (HRTEM) operated at 400 kV characterized by a spatial resolution by points to point of 0.16 nm and by lines of 0.1 nm. The planar and cross-sections for HRTEM analysis were prepared in a standard manner by etching [31] with argon ions. SingleCrystalTM and CrystalMaker^®^ were used [32] to determine the observed zone axis of the TEM images. Digital Micrograph (GATAN) was used for digital processing of the experimental HRTEM images. 

### 2.4. Magnetic Measurements

The magnetic properties of the samples were studied using a vibrating magnetometer, which is part of the Quantum Design PPMS 9T ECII system (Quantum Design International, San Diego, CA, USA) for measuring the physical properties. The field dependences were obtained at two temperatures, namely 300 K and 2 K, and a field range of ±30 kOe for three directions of the magnetic field. We chose two directions of the magnetic field along the plane of the substrate (along and across the NRs) and one direction perpendicular to the plane of the substrate and plane of the NRs.

FORC studies [33,34,35] were performed on a LakeShore VSM 7410 vibrating magnetometer at room temperature. To this end, a set of 100 magnetization reversal curves were measured in an external field directed along the NRs in a field range of ±1 kOe with a step of 20 Oe. After obtaining a set of experimental curves, the data were processed using the doFORC software package [36].

### 2.5. Ab Initio Calculations of Magnetic Moments in Nanowires 

Using the ab initio total-energy projector-augmented wave method (code VASP-Vienna Ab initio Simulation Package, version 5.4.4) [37,38,39,40] within the generalized gradient approximation (GGA) of Perdew–Burke–Ernzerhof (PBE) [41], we investigated the appearance of magnetic moments on Fe atoms in freestanding, <110>-oriented α-FeSi_2_ NWs. Such NWs were considered a periodic arrangement of wires with 12 Å of vacuum to ensure negligible interaction between neighboring NWs. An energy cutoff of 380 eV and grid of 1 × 1 × 4 Monkhorst–Park points were used in the calculations. Atomic relaxation was stopped when the forces on the atoms were smaller than 0.03 eV/Å. To ensure convergence with respect to the *k*-points, the final iterations were performed on a 1 × 1 × 7 grid.

## 3. Results

### 3.1. Morphology and Crystal Structure of α-FeSi_2_ Nanorods on Si(111)-4° Surface

**Figure 1:**
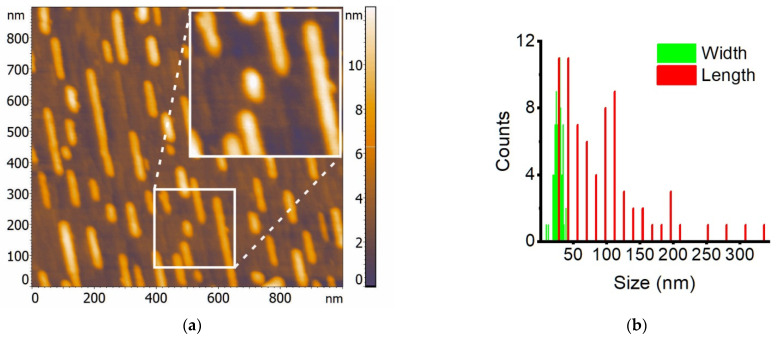
(**a**) AFM image of α-FeSi_2_ nanorods on a Si(111)-4° vicinal surface; a square with a white border is highlighted and its enlarged image is shown in the inset; (**b**) size distribution of nanorods.

**Figure 2:**
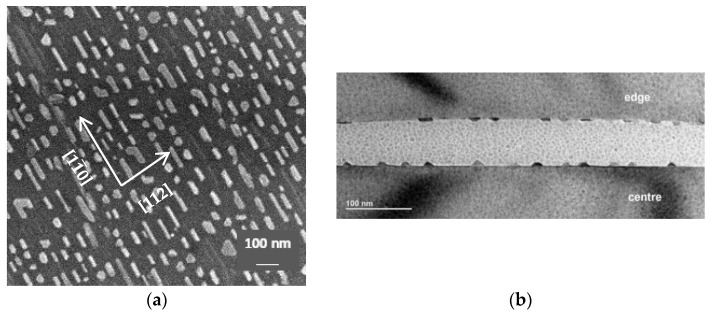

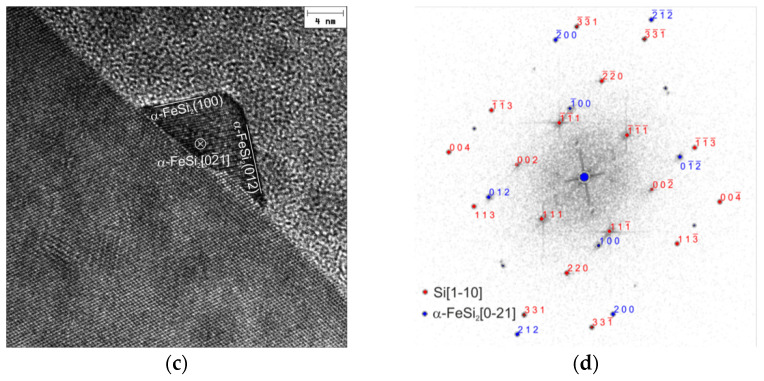
TEM (planar) and HRTEM on cross section images of Fe silicide/Si interfaces: (**a**) general view of two glued samples; (**b**) cross section of Fe silicide NR with its main facets and direction [0$\bar{2}$1]; (**d**) FFT pattern from areas (**c**) with identification of α-FeSi_2_ planes.

After unloading the sample from the UHV chamber, its surface morphology was studied by AFM. In accordance with the AFM data [27] and taking into account slightly smaller initial iron coverage in our case, the density and size of the islands were expected to be smaller. In fact, according to AFM data (Figure 1a), intergrown islands were formed in a form of self-ordered NRs with density of 1.0 × 10^10^ cm^−2^ and located along step-bunches, as reported in [2,3,4,27]. Processing of AFM images using a specialized program [30] made it possible to calculate the statistical characteristics of the obtained array of NRs. The distribution of NR sizes is shown in Figure 1b. The average width, height, and aspect ratio of the length to width of NRs were calculated to be 30 nm and 6.6 nm, and 3.5, respectively. Average values and standard deviation of NRs lengths and widths (103 ± 67 nm and 28 ± 5 nm, respectively) was calculated from the data in Figure 1b. From the obtained data on the NRs, their average volume was calculated by assuming invariable composition for all NRs. Since the FeSi_2_ stoichiometry was confirmed by HRTEM and fast Fourier transform (FFT) data (Figure 2c,d), calculations of the initial iron amount to from NRs were carried out. It was 0.482 nm in thickness, which corresponds to 5.22 MLs; note that 1 ML of Fe on a Si(111) surface has a density of 7.83 × 10^14^ atoms/cm^2^. The obtained average value of the iron coating in the experiment was in good agreement with the calibration data for a quartz thickness gauge and is in the range specified above according to the work data: 1.2–21.0 MLs [2]. It can be clearly seen in the inset in Figure 1a, that the NRs can be viewed as fused (coagulated) islands with sizes ranging from 20 to 30 nm in length and width. NRs up to 300 nm long were oriented predominantly in one direction owing to the direction of step-bunches on the vicinal surface after high-temperature annealing. The image also shows individual islands in the vicinity of the NRs, which indicates the absence of intense coagulation by the Ostwald ripening mechanism at the annealing temperature (630 °C). When a thin layer of iron is annealed, it fragmented into separate islands, followed by surface diffusion of iron atoms across step-bunches and, eventually, silicidation. Such diffusion of iron atoms was previously discovered for temperatures ranging from 450 to 550 °C with a noticeably lower iron coverage (1.2 MLs) [2] without considering both a higher iron coverage (21 MLs) and annealing (700 °C). Thus, the self-formation of ordered NRs is the main mechanism for the growth of the silicide phase during the deposition of iron coverage of approximately 5 MLs on the Si(111)-4° vicinal surface.

The surface morphology and crystal structure of the NRs were further studied using TEM and HRTEM on the planar and transverse sections of the sample after thinning (Figure 2). Figure 2a shows (1) a planar image of the NRs as individual islands, which correlates well with the AFM data (Figure 1a), and (2) the predominant location of the islands along the step-bunch edges and with [0$\bar{2}$1] orientation of their cross-section (Figure 2c). A general view of the cross-section of the two halves of the sample is shown in Figure 2b, with both parts of the cross-section taken from the central part of the sample. It can be seen that the NRs intersect perpendicularly with the image plane. An analysis of the FFT pattern of the NR sections (Figure 2c) revealed a triangular shape and a structure of the alpha phase of iron disilicide (α-FeSi_2_) with the tetragonal crystal lattice. In addition, it can be seen that the NRs were attached to a step-bunch with a height of approximately three silicon MLs, which caused a slight tilting with respect to the terrace surface with the (111) orientation (Figure 2c). Checking the images of NRs at several points resulted in the same picture. It should be noted that there was no transition layer at the interface with silicon in agreement with the data reported in [27]. This indicates the absence of diffusion of excess Fe atoms into silicon and silicidation proceeds mainly owing to the diffusion of Si atoms (also from the step-bunches) into the deposited Fe layer during solid-phase annealing. However, during annealing at 550 °C a transition layer was noticeable on the HRTEM of the cross-section of α-FeSi_2_ NRs as reported in [2]. The FFT of the local area in Figure 2c, which captures the cross-section of the NR, is shown in Figure 2d along with the interpretation of the main planes of silicon and α-FeSi_2_ phase. NRs are characterized by the following epitaxial relationships: α-FeSi_2_(112)//Si(111), for which there is a small misorientation angle (which correlates with the data in Figure 2c), and α-FeSi_2_[0$\bar{2}$1]//Si[1$\bar{1}$0]. The calculations of the parameters of the crystal lattice in the case of α-FeSi_2_[0$\bar{2}$1]//Si[1$\bar{1}$0] showed that the parameter “a” was compressed by 0.55% and presented a value of 0.26752 nm. In this case, parameter “c” was stretched by 7.69% and had a value of 0.54709 nm. In general, this led to a 6.5% increase in the unit cell volume of α-FeSi_2_ for NRs compared to that of the relaxed unit cell of α-FeSi_2_. Thus, the non-ideal conditions of epitaxial relationship of the α-FeSi_2_ lattices and Si(111)-4° vicinal surface with step-bunches lead to tensile stresses. 

### 3.2. Magnetic Hysteresis Loops of a System of α-FeSi_2_ Nanorods on the Vicinal Si(111)-4° Surface 

**Figure 3:**
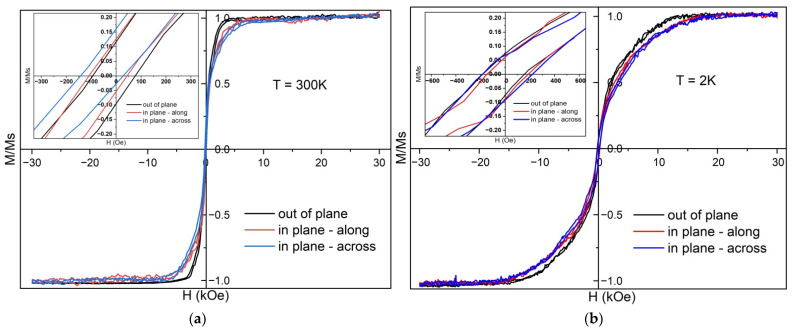
Magnetization reversal loops of the α-FeSi_2_ NRs array on Si(111)-4° at 300 K (**a**) and 2 K (**b**) for three directions of the magnetic field (“out of plane”; “in plane—along”; “in plane—across”). The insets show the area of small fields.

The magnetic properties of the grown NRs with α-FeSi_2_ structure were studied at two temperatures: 300 K and 2 K. The diamagnetic contribution of the silicon substrate was subtracted from the measured magnetization signal of the sample with silicide NRs. Subsequently, the magnetic moment (M) was normalized to the saturation magnetic moment (Ms). The measurements showed that the ferromagnetic nature of the magnetization of the silicide NRs extended in three directions of the magnetic field (in the plane of the sample: along and across the NRs or, abbreviated, “in-plane-along” and “in-plane-across,”, respectively) and perpendicular to the plane of the sample (“out-of-plane”), both at 300 K (Figure 3a) and at 2 K (Figure 3b). The absolute values of the saturation magnetic moment of the NRs were approximately 7.5 μemu at 2 K and 2.2 μemu at 300 K, and their coercive force (H_C_) increased with decreasing temperature from 90 Oe to 180 Oe (Figure 3a,b, left and right insets). In this case, the values of *H_C_* for the three directions of the magnetic field (MF) did not differ at any temperature. This is inconsistent with the strong dependence of *H_C_* on the temperature and direction of the magnetic field along the plane of the α-FeSi_2_ NRs formed with an initial iron coverage of 1.2 MLs [2]. The weakening of the coercive force in the direction of the MF perpendicular to the substrate and NR system was reported in [2] because of defects enriched in iron atoms with a strong ferromagnetic bond that line up during formation mainly along the direction of the NRs but not perpendicular to them. The latter assumption may be related to the predominant topology of NRs in [2], which appeared continuous along the length. In our case, the NRs are in the form of coagulated islands with clearly visible boundaries (Figure 1a, inset) and an average height of 6.6 nm. This generally agrees with the planar TEM image data (Figure 2a) where a flat surface can be observed, whereas the AFM image also provides information about height.

At room temperature, for an MF perpendicular to the substrate and NRs, the saturation magnetic moment was occurred at 4 kOe, whereas for an MF parallel to the NRs, the magnetic moment saturated only at 9 kOe. Such anisotropy with the direction of the easy magnetization axis perpendicular to the plane of the substrate may be due to stresses in the NRs (Figure 2d). For a more detailed study of the magnetic anisotropy of NRs, it is necessary to carry out angular measurements of their magnetic properties in steps of approximately 5°.

A decrease in temperature to 2 K led to a significant increase in MFs at which saturation of the magnetic moment was formed: 13 kOe for an MF perpendicular to the NRs and 20 kOe for a magnetic field along the NRs. When the sample was cooled to 2 K, the resulting magnetic moment (RMM) increased by a factor of 3.4 approximately, regardless of the direction of the MF. Such a strong drop in the magnetic moment with increasing temperature indicates either the proximity of the Curie point or additional processes that affect the magnitude of the magnetic moment, in addition to a linear decrease in RMM with increasing temperature. 

Although the shapes of the hysteresis reversal loops obtained at different temperatures (Figure 3) were notably different, an inflection point can be observed between the two linear sections in both cases. Such loops with inflection are typical of systems with two magnetic phases having different saturation fields. According to the AFM analysis (Figure 1a), NRs with significantly different lengths and heights, and consequently, different volumes, were observed, and their sizes were characteristic of the superparamagnetic state [42,43]. In such systems, the height of the energy barrier separating two stable states along the easy axis of a single-domain nanoparticle is determined by the value of uniaxial effective anisotropy *K_eff_* (magnetocrystalline anisotropy, shape anisotropy, or their combination) and the nanoparticle volume *V* [44]:

*E_b_* = *K_eff_V* (1)

If the energy of thermal fluctuations, *k_B_T*, is below this energy barrier, the particle will exist in a single-domain ferromagnetic state. When the Curie temperature is reached and this energy barrier is exceeded, the nanoparticles pass into the paramagnetic state. If *k_B_T* is comparable to the energy barrier height, then SPM is observed in the nanoparticles. This phenomenon is described as a constant switching of the magnetization in the nanoparticles during the Néel relaxation time *τ_N_*:

(2) $$\tau_{N}=\tau_{0}exp\left(\frac{K_{eff}V}{k_{B}T}\right)$$

where *τ*_0_, which is characteristic of the material, is defined as the time interval during which magnetization tries to switch. 

Owing to the sizable difference in the volumes of the NRs, it can be assumed that our sample contained both nanostructures in the single-domain state and displaying superparamagnetic and paramagnetic properties. Each of these magnetic phases interacts differently with an external magnetic field. It is possible to omit the influence of the paramagnetic phase on the hysteresis reversal loops, since it possesses the linear dependence of the magnetization on the magnetic field. Consequently, it can be assumed that two phases, namely ferromagnetic and superparamagnetic, are simultaneously remagnetized on loops in low fields up to the inflection point. At the inflection point, one of these phases reaches saturation, whereas the second continues the magnetization until the sample is completely saturated. With an increase in temperature, the ratio of the ferromagnetic and superparamagnetic phases changes because some of the nanoparticles with small volumes pass from the superparamagnetic state to the paramagnetic state, and some of the ferromagnetic nanoparticles pass from the single-domain state to the superparamagnetic state leading to the change in the shape of the loops. Large saturation fields at low temperatures can be explained, among other causes, by the strong magnetostatic interactions of a large number of densely located magnetic segments with a high magnetic moment. As the temperature rises to room temperature, some of these structures pass into a paramagnetic state, the distance between the magnetic neighbors increases, and the magnetic moment of the magnetic structures decreases due to temperature fluctuations. As a result, the interaction fields decrease significantly.

### 3.3. Determination of the Domain Composition of α-FeSi_2_ Nanorods by the FORC-Diagram Method

FORC diagrams have been used to study various systems of magnetic particles [33,34,35]. To build FORC diagrams, a set of partial hysteresis loops is experimentally recorded. The measurement begins with sample saturation in an external magnetic field (*H_sat_*). In addition, the applied field decreases to the inversion field *H_a_*, and then increases back in the applied field *H_b_* to saturation, where the FORC magnetization curve ends. A set of partial hysteresis loop curves is obtained by repeating the measurements for different values of *H_a_*. The FORC distribution is defined as the mixed second derivative *ρ*(*H_a_*, *H_b_*) with respect to the variables *H_a_* and *H_b_* [33]. The FORC diagram is a FORC contour plot of the distribution of *ρ*(*H_a_*, *H_b_*) along the *H_C_* and *H_U_* axes, where the coordinates along the horizontal

*H_C_* = (*H_b_* − *H_a_*)/2 (3)

and vertical

*H_U_* = (*H_b_* + *H_a_*)/2 (4)

axes are taken from [33].

**Figure 4:**
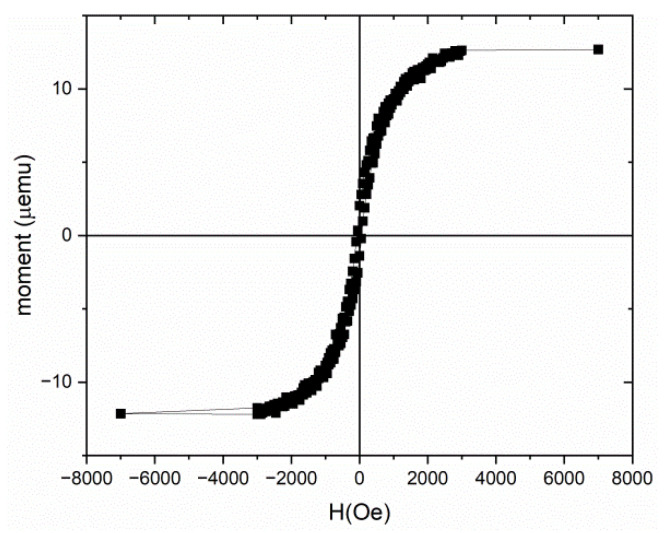
Hysteresis loop along the α-FeSi_2_ NRs on the Si(111)-4° surface with the linear diamagnetic slope subtracted from the silicon substrate.

At the beginning of the experiments, a hysteresis reversal loop was recorded at room temperature by subtracting the diamagnetic contribution from the silicon substrate (Figure 4). The obtained values of the saturation magnetic field were in good agreement with the measurement data presented in Figure 3a, and the discrepancy in magnetic moments is associated with the different sizes of the samples used, since samples to collect the data in Figure 3 were limited by the maximum possible size to be placed in a special holder. Subsequently, in accordance with the method presented above, partial hysteresis reversal loops were measured with 100 curves and a saturation field *H_sat_* = 1000 Oe with a measurement step *H_r_* = 20 Oe. Due to the fact that the measured magnetic moments were very small and close to the sensitivity limit of the vibromagnetometer owing to high noise, the signal accumulation time was chosen to be 10 s. Next, FORC diagrams were constructed in the direction of the field parallel to the α-FeSi_2_ NRs (Figure 4).

**Figure 5:**
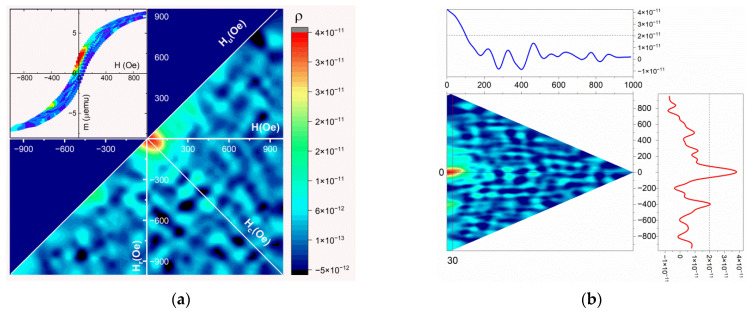
(**a**) Resulting FORC-diagram with an averaging factor of 100 for a system of α-FeSi_2_ nanorods on a Si(111)-4° surface; a set of FORC curves is presented in the inset. The *ρ* value is color-coded; (**b**) rotated FORC-diagram and profiles along *H_U_* and *H_C_* axes.

After obtaining the FORC curves, they were processed using the do FORC software package [36], in accordance with the formula for *ρ*(*H_a_*, *H_b_*) reported in [33]. Subsequently, a square grid was built with different averaging coefficients *n* (50 and 100) for *ρ*(*H_a_*, *H_b_*), taking into account the method of numerical estimation of the optimal value of n [43]. Figure 5a shows a FORC diagram with an averaging factor of *n* = 100 and a set of FORC curves (inset) along with the value of the second derivative *ρ* which is in color [33]. Blue and green indicate the minimum *ρ* whereas red stands for the maximum *ρ* (Figure 5a). The diagram [33] rotated for the transition to the axes *H_C_* (formula (3)) and *H_U_* (formula (4)) FORC. Its profiles along the corresponding axes are shown in Figure 5b. Despite the high noise in the FORC diagram, even after smoothing procedures, we can clearly distinguish the main peak with a high maximum of *ρ* in the region in which the values of *H_U_* and *H_C_* are close to zero. Analysis of the FORC-diagram profiles showed weakly interacting single-domain grains related to α-FeSi_2_ NRs characterized by critical fields of the coercive force *H_C_* ranging from 0 to 130 Oe. They were not shifted along the *H_U_* axis, according to theoretical concepts [33,34,35] of single-domain objects in ferromagnetic systems. The FORC diagram confirms the assumption about practically non-interacting NRs with different volumes and, consequently, the validity of the coercive force estimates in Section 3.2. To confirm this assumption more accurately, in future work, we will measure the low-temperature FORC diagram of this sample.

### 3.4. Ab Initio Calculations of Magnetic Moments in Freestanding α-FeSi_2_ Nanowires

**Figure 6:**
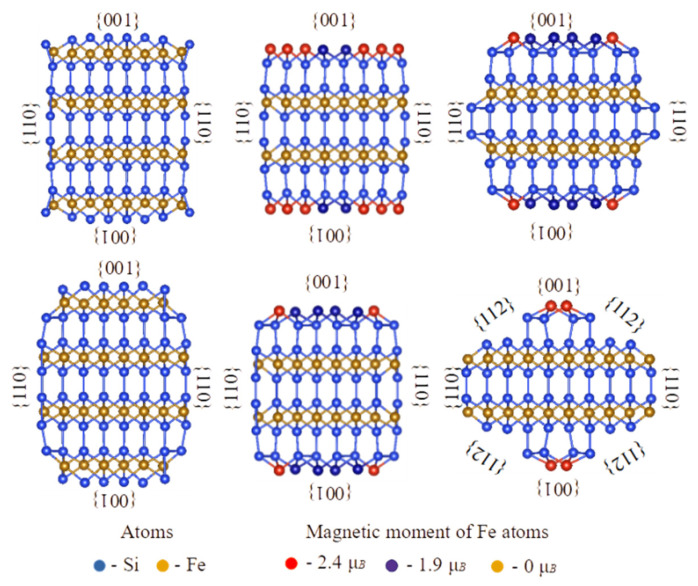
Cross sections of <110>-oriented α-FeSi_2_ NWs with different morphologies indicating surface Fe atoms with magnetic moments. All facets are shown.

**Figure 7:**
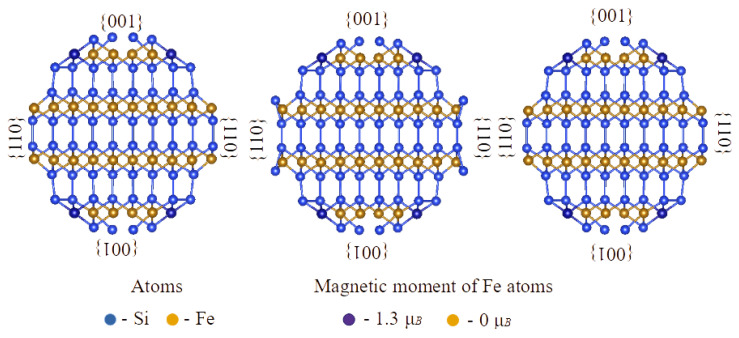
Cross sections of <110>-oriented α-FeSi_2_ NWs with the different morphologies indicating surface Fe atoms with magnetic moments. All facets are shown.

**Figure 8:**
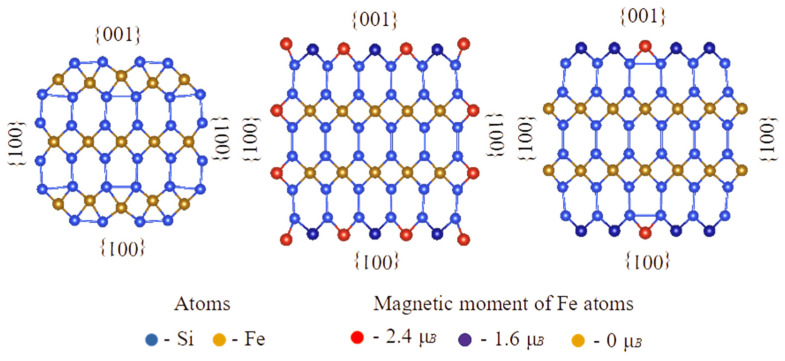
Cross sections of <100>-oriented α-FeSi_2_ NWs with different morphologies indicating surface Fe atoms with magnetic moments. All facets are shown.

It is difficult to consider experimentally observed morphology of α-FeSi_2_ nanostructures on Si(111) by using *ab initio* methods, since the interface with the substrate should be taken into account. In addition, if the α-FeSi_2_ bulk had the cubic symmetry instead of the tetragonal one, it would be possible to construct a unit cell keeping the orthorhombic symmetry in order to model a α-FeSi_2_(012) surface and a <0$\bar{2}$1>-oriented α-FeSi_2_ NW. In our case, to consider the latter one with experimentally observed {100} and {012} facets (according to Figure 2c), the size of the unit cell was found too large to afford calculations in addition to triclinic symmetry lowering (angles between the cross-section plane and facets on the surface are not 90°). That is why we investigated α-FeSi_2_ NRs as freestanding NWs with <100> and <110> orientations and different facets on the surface (see Figure 6, Figure 7 and Figure 8). The morphology of an NW provides a good opportunity to vary facets ({001}, {100}, {110} and {112} in our case) as well as the coverage of Si and/or Fe surface atoms and introduce edges between adjacent facets (similar to the case of Si NWs [45]). Moreover, the (012) surface can be viewed as a stepped surface with (010) or (100) steps. This should shed some light on the appearance of magnetic moments on atoms with respect to morphology in α-FeSi_2_ nanostructures. In fact, we traced only surface Fe atoms on {001} and {100} facets (Figure 6 and Figure 8) and on edges between {110} and {001} facets (Figure 7) to have magnetic moments, whereas Fe atoms on {110} facets and inside the NWs did not possess any magnetic moment. The observed magnetic moments, in the range 1.3–1.9 Bohr magnetons/Fe atom, are typical of surface Fe atoms with a lack of some Si atoms as the first neighbors, whereas magnetic moments of 2.4 Bohr magnetons/Fe atom occurred when surface Fe atoms were too close to each other (compare 2.7 Å in bulk and ~2.3 Å at the surface for Fe–Fe interatomic distances), indicating the formation of chemical bonds. These calculations have been performed for α-FeSi_2_ NWs with diameters of about 2 nm, whereas some test calculations for 4 nm diameter NW revealed the same tendencies in the appearance of magnetic moments of surface Fe atoms.

## 4. Discussion

A review of experimental and theoretical studies on the magnetic properties of nanosized semiconductor [5,6,12,25,26], metallic, and semimetallic iron silicide [1,2,3,4] (nanoislands, NRs, NWs) that do not have magnetic properties in the bulk shows that they exhibit superparamagnetic properties at very low temperatures (down to helium ones). At room temperature, a transition to paramagnetic properties occurs most often, which makes it possible to obtain different temperature values to block paramagnetic properties in such nanostructures depending on their size [2]. In this case, the manifestation of magnetic properties for all nanostructures is associated either with the appearance of edge iron atoms with spin moments, with the incorporation of iron atoms into the interstices of the iron silicide lattice, or with the multi-phase nature of the system under study and the appearance of inclusions of iron clusters with ferromagnetic properties. However, for the considered iron silicides in the form of nanoislands, NRs, and NWs of any composition with a small iron coating [1,2,3,4] added initially, during chemical processes [5,6], or through laser ablation [26], the ferromagnetic properties were not preserved up to room temperature. However, maintaining ferromagnetic properties down to room temperature is critical for super-dense magnetic memory when using such nanostructures, which are self-ordered on silicon. 

In this study, during solid-phase epitaxy at a temperature of 630 °C and iron coverages (5–6 MLs) the self-formation of α-FeSi_2_ NRs with a unit cell increased in volume by 6.5% due to heteroepitaxial growth on silicon has been traced along with soft ferromagnetic properties up to 300 K and the disappearance of the blocking temperature typical of nanostructures obtained at smaller iron coverages (1.2 MLs) [2]. A legitimate question arises: What is the nature of ferromagnetism in these NRs? The study of the field dependences of the magnetic moment in α-FeSi_2_ NRs at two temperatures (2 K and 300 K) and three directions of the magnetic field (“in-plane-along”, “in-plane-across” and “out-to-plane”) showed (see Figure 3a,b) that the saturation of the magnetic moment occurs faster for high temperatures. At room temperature, for the “out-of-plane” direction of the magnetic field, this process was observed at 4 kOe, and at *T* = 2 K, it was observed at 13 kOe. The measurements also showed that the easy magnetization axis was directed perpendicular to the α-FeSi_2_ NRs. The hard magnetization axis lies in the “in-plane” direction with saturation at a magnetic field of 9 kOe at 300 K and 20 kOe at 2 K. This differs from the case with a small iron coverage (1.2 MLs) [2], for which the easy magnetization axis is parallel to the direction of the NRs, which is explained by the location of defects enriched in iron atoms along the NRs. In our samples, the situation was different. In NRs, the concentration of iron atoms in the cross-section of the α-FeSi_2_ NRs is somewhat higher than that along and across the NRs on their surface. In other words, there is a preferential distribution of iron atoms deep into the NRs. The distribution “in-plane” is associated with iron atoms near the steps and kinks, as theoretically proven [8]. Taking into account the coagulation of the islands in our case, some of the iron atoms are highly probable to diffuse to the substrate and can be distributed over the thickness of the NRs, and some other propagate along their surface, as reported in [2]. The ability for bulk diffusion of unreacted iron atoms into NRs and their incorporation into the interstices of the α-FeSi_2_ crystal lattice was confirmed by the data on the α-FeSi_2_ crystal lattice collected in this study through HRTEM (Figure 2d). Note that there is a noticeable stretching along the “c” axis and an increase in the unit cell volume of α-FeSi_2_ up to 6.5% due to heteroepitaxial incorporation into the silicon lattice. The RMM value calculated at 2 K from the absolute value of the magnetic saturation moment of the NRs (Figure 3a) and reduced to the effective iron coverage deposited at room temperature on a silicon substrate (from AFM data, Figure 1a,b) resulted in 2.38 Bohr magnetons/Fe atom. This RMM value is slightly higher than that for pure iron, 2.2 Bohr magnetons/Fe atom [9], and for α-FeSi_2_ NRs, 1.9 Bohr magnetons/Fe atom, with an iron coverage of 1.2 MLs [2], thereby demonstrating a strong ferromagnetic bond (exchange interaction) between the iron atoms in our sample in two directions (“in-plane” (along and across) and “out-of-plane”). As the temperature increased to 300 K, the RMM decreased to approximately 0.7 Bohr magneton/Fe atom, which is associated with the thermal misorientation of spin magnetic moments, a decrease in spontaneous magnetization, and a weakening of the contribution of the exchange interaction. At zero magnetic field, the remanence magnetization (0.5–0.6 μemu) at a temperature of 2 K (Figure 3b, inset) is practically independent of the magnetic field direction (parallel or perpendicular to the substrate and system of NRs). A similar trend was observed at room temperature. The values of the squareness coefficient were S_n_ = 0.073–0.075 at 2 K and S_n_ = 0.045–0.050 at 300 K, which confirms the low anisotropy of the system. In other words, α-FeSi_2_ NRs with an average thickness range over the iron coverage (5.22 MLs) exhibit soft ferromagnetic properties with weak magnetic anisotropy, a threefold decrease in the RMM value, and a twofold decrease, approximately, in the coercive force (Figure 3a,b) when heated from 2 K to 300 K. This is promising for the formation of magnetic memory with a high density specified in the process of self-formation within planar silicon technology. 

The magnetic hysteresis loops obtained at different temperatures have two linear sections with different slopes separated by a kink, which is typical of the presence of two magnetic phases with different saturation fields in the system under study. According to these assumptions, these phases can be large-volume single-domain ferromagnetic NRs or small-volume superparamagnetic particles. An increase in temperature leads to a change in the ratio of these two phases as well as a decrease in the magnitude of magnetostatic interactions, which in turn provides a change in the saturation fields and shape of the hysteresis loop.

The stable ferromagnetic properties of nanostructures are determined by their magnetic domain structures. However, owing to the small width of the α-FeSi_2_ NRs (20–30 nm), large radius of curvature of the cantilever with magnetic coating (approximately 20 nm), small magnetic moment per NR (small fractions of μemu units) at low fields and room temperature, magnetic force microscopy (not shown in this paper) failed to register the magnetic contrast in individual NRs and determine their magnetic domains. To solve the problem of the nature of magnetic domains, a method was used to measure partial hysteresis loops and construct FORC diagrams according to the method reported in [33,34,35]. The constructed FORC diagram with averaging coefficient *n* = 100 (Figure 5b) shows that in α-FeSi_2_ NRs, owing to their spread in size (length, width, and height) (Figure 1a,b and Figure 2a–c), only one region was formed. This region was determined by the range of coercive forces *H_C_* 0–130 Oe with a small interaction field (*H_U_*). This corresponds to the presence of weakly interacting ferromagnetic regions in the α-FeSi_2_ NRs, given that the silicon substrate itself is diamagnetic.

*Ab initio* calculations of the magnetic moments in freestanding α-FeSi_2_ NWs with the same orientation and different NR cross-sectional areas (facets) showed that the magnetic moment arises only when iron atoms terminate the α-FeSi_2_(001) surface, and its average value depends on the number of iron atoms in the NR section (Figure 6 and Figure 7). This agrees with the data of first-principle theoretical calculations of the magnetic moment reported in [9] when the surface of the FeSi(111) layers is terminated by iron atoms. However, in contrast to a previous study [9], when the surface of α-FeSi_2_(100) was terminated by silicon atoms, no magnetic moment was formed in the NRs of α-FeSi_2_. Only qualitative comparison with experimental data is possible because the real epitaxial relationship with the silicon lattice, shape of the crystallized NRs, and stresses were not taken into account. Ab initio calculations were carried out at 0 K, so the results could only be compared with the magnetic moment at 2 K. As mentioned above, the resulting magnetic moment calculated at saturation in a magnetic field was 2.38 Bohr magnetons/Fe atom, which is in good agreement with the theoretical data for NWs with maximum section size for individual atoms in the {100} facets: 2.4 and 1.9 Bohr magnetons/Fe atom (Figure 6). However, when recalculating the RMM with the number of iron atoms in the NW cross-section, we unavoidably obtain a RMM with a sizably smaller value (also taking into account that with an increase in diameter the amount of Fe surface atoms decreases with respect to the amount of bulk-like Fe atoms). The difference can arise from the fact that in our experiments, either atoms or defects enriched in iron atoms on the surface of the NRs and, in a larger amount, iron atoms embedded in the bulk of the α-FeSi_2_ NR crystal lattice, make a contribution. 

## 5. Conclusions

In this study, α-FeSi_2_ NRs were grown on a vicinal Si(111)-4° surface. The size distribution of the NRs (width and length) and height was established, as well as the average coverage of iron atoms (5.22 MLs) was calculated to ensure their self-ordering on the vicinal silicon surface along the step-bunches. The α-FeSi_2_ NRs demonstrated soft ferromagnetic properties (coercive force from 90 to 180 Oe), which were preserved in the temperature range from 2 K to 300 K, with an easy magnetization axis perpendicular to the NRs and a hard magnetization axis parallel to the NRs caused by elastic stresses. The analysis of the hysteresis loops showed the presence of two phases with different saturation fields. An increase in temperature led to a change in the ratio of these phases and a decrease in the magnitude of magnetostatic interactions. It has been found that the contribution of bulk iron atoms in the structure of α-FeSi_2_ NRs to the magnetic moment slightly exceeds the contribution of surface iron atoms along the NRs, thereby reducing the anisotropy of their magnetic properties compared to self-ordered islands of α-FeSi_2_. However, due to this phenomenon, the preservation of ferromagnetism in the system of NRs is affected. Thus, the nature of ferromagnetism lies in the preservation of the volume and surface contributions of iron atoms or iron-rich defects in self-ordered α-FeSi_2_ NRs. The formation of weakly interacting ferromagnetic α-FeSi_2_ NRs with different coercive forces in the range of 0–130 Oe due to a strong scatter over their volume was confirmed by the FORC-diagram method. Ab initio calculations of magnetic moments in freestanding α-FeSi_2_ NWs confirmed the formation of spin moments for Fe atoms only at the {001} facets and on edges between {110} and {001} facets. If an averaged magnetic moment assuming all Fe atoms in the cross section of a NW is calculated, its value is smaller than the experimental value of 2.38 Bohr magnetons/Fe atom due to possible contributions from defects enriched in Fe atoms on the surface of the NRs and Fe atoms acting as an interstitial impurity in the bulk of the α-FeSi_2_ NR crystal lattice. Further calculations are planned to be carried out considering the epitaxial relationship of the NWs with the silicon substrate. 

## Data Availability

The data presented in this study are available upon reasonable request from the corresponding author.
